# Prognostic Value of MTV and TLG of ^18^F-FDG PET in Patients with Stage I and II Non-Small-Cell Lung Cancer: a Meta-Analysis

**DOI:** 10.1155/2021/7528971

**Published:** 2021-11-22

**Authors:** Weibo Wen, Yongnan Piao, Dongyuan Xu, Xiangdan Li

**Affiliations:** ^1^Department of Nuclear Medicine, Yanbian University Hospital, Yanji 133000, China; ^2^Center of Morphological Experiment, Medical College of Yanbian University, Yanji 133000, China

## Abstract

**Purpose:**

The present systematic literature review and meta-analysis focused on examining the significance of total lesion glycolysis (TLG) and metabolic tumor volume (MTV) in predicting the prognosis of stages I/II non-small-cell lung cancer (NSCLC) based on ^18^F-FDG PET parameters.

**Methods:**

Electronic databases, including Cochrane Library, PubMed, and EMBASE, were comprehensively searched for retrieving relevant articles published in the English language. Furthermore, the significance of TLG and MTV in prognosis prediction was analyzed by pooled hazard ratios (HRs).

**Results:**

This work enrolled eight primary studies with 1292 I/II-stage NSCLC cases. The pooled HR (95% confidence interval [CI]) for the ability of increased TLG to predict progression-free survival (PFS) was 2.02 (1.30–2.13) (*P*=0.350), while for increased MTV it was 3.04 (1.92–4.81) (*P*=0.793). In addition, the pooled HR (95% CI) for the ability of increased TLG to predict overall survival (OS) was 2.16 (1.49–3.14) (*P*=0.624). However, higher MTV correlated with OS, and sensitivity analysis showed that the results were not stable. Multivariate and univariate analyses by subgroup analyses stratified by PFS of MTV and OS of TLG exhibited statistically significant differences, without any statistical heterogeneity across various articles.

**Conclusion:**

The present work suggests the predictive value of PET/CT among stage I and II NSCLC patients. Our results verified that stage I/II NSCLC cases with increased TLG and MTV had a higher risk of side reactions, and TLG is related to increased mortality risk.

## 1. Introduction

Non-small-cell lung cancer (NSCLC) represents a frequently occurring lung cancer subtype, with its incidence rising globally [1]. It is still responsible for most cancer-related deaths worldwide [2, 3]. Accurate prognostic factors are essential for patient management, as patients with surgery or dismal prognosis can benefit from additional neoadjuvant treatment [4].

More attention has been paid to applying the volumetric metabolic parameters like metabolic tumor volume (MTV) or total lesion glycolysis (TLG). The average SUV and MTV are determined through the threshold-defined margin contouring. TLG is determined by the multiplication of MTV with average SUV, and it can weigh tumor metabolic activity and volumetric burden [5–7]. TLG and MTV from 18F-fluorodeoxyglucose (FDG) positron emission tomography/computed tomography (PET/CT) have been identified as the standard staging methods, also used to monitor therapeutic response and predict prognosis of different cancers, such as NSCLC [5, 8–10]. As suggested in recent systematic reviews and meta-analyses [11, 12], TLG and MTV negatively correlated with NSCLC prognosis. Consequently, it is essential to identify prognostic factors for NSCLC cases [13].

Some articles examined the relationships of tumor prognosis and response with TLG and MTV from 18F-FDG PET in stage I/II NSCLC patients. Nonetheless, the significance of TLG and MTV from 18F-FDG PET/CT for the prognosis prediction of stage I/II NSCLC patients remains controversial. Certain articles suggested that the increased MTV was significantly related to the dismal prognostic outcome for NSCLC patients in stages I and II [14, 15]. In contrast, a different conclusion was observed by Vu et al. [16].

In this regard, the present meta-analysis focused on summarizing findings reported in published articles examining the significance of TLG and MTV in predicting progression-free survival (PFS) and overall survival (OS) in stage I/II NSCLC patients.

## 2. Materials and Methods

### 2.1. Study

The present study was carried out following the preferred reporting items of the systematic review and meta-analysis (PRISMA) statement guidelines [17].

### 2.2. Data Search and Study Selection

Electronic databases, including Cochrane Library (2012–May 2019), PubMed, and Embase, were searched using the keywords below (“NSCLC” OR “lung neoplasms” OR “lung carcinoma” OR “lung neoplasms”) AND (“positron emission tomography-computed tomography” OR “PET-CT” OR “positron emission tomography-computed tomography” OR “PET/CT” OR “positron emission tomography” OR “PET CT” OR “fluorodeoxyglucose” OR “FDG”) AND (“outcome” OR “prognosis” OR “prognostic” OR “survival” OR “predictive”). Studies conforming to the following criteria were included: (1) studies including the histological diagnosis of stage I and II NSCLC patients; (2) studies using 18F-FDG PET/CT as the imaging modality prior to treatment, articles that reported survival data by MTV or TLG; (3) articles published in English. However, case reports, reviews, editorial materials, and conference abstracts were excluded. Studies were searched and screened by two independent reviewers, and any disagreement between them was settled through mutual negotiation to reach a consensus.

### 2.3. Statistical Analysis

The identical method utilized in our prior work was adopted [18]. The present work pooled disease-free survival (DFS), recurrence-free survival (RFS), and PFS from all the enrolled articles and redefined PFS [19]. Parmar et al.'s method was adopted for extracting survival data [20]. PFS, OS, hazard ratios (HRs), and the appropriate 95% confidence intervals (95% CIs) with corresponding variations were determined through STATA version 12.0 (STATA Corp., College Station, TX). Data on HRs and 95% CIs obtained by multivariate analysis were obtained directly from each work. As for missing multivariate HRs, the univariate HRs were obtained. For missing univariate and multivariate HRs, Parmar et al.'s method [21] was adopted for reconstructing HR estimates together with the variance using Kaplan–Meier curves-derived survival data through Engauge Digitizer (version 9.4). The pooled HR represented the effect value displaying the significance of prognosis. HR > 1 indicated a poor prognosis for cases showing increased TLG or MTV, while HR < 1 stood for survival benefit for cases showing increased TLG or MTV. Egger's test and Begg's test were adopted for evaluating bias using STATA version 12.0.

## 3. Results

### 3.1. Search Results

**Figure 1:**
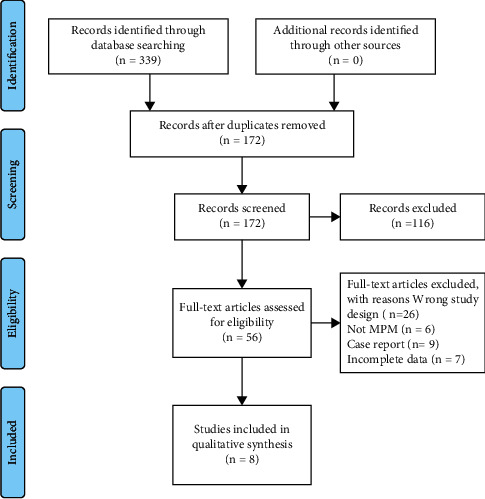
Flowchart of our study selection.

Our study searched electronic databases Embase, Cochrane Library, and PubMed, and 177, 0, and 162 studies involving 1,590 cases were collected, respectively. Meeting summaries and duplicates were excluded, and 56 eligible studies were retained. Among them, 48 were eliminated, including 26 due to unwanted study design, six unrelated to NSCLC, 9 introducing one case report, and seven without creditable data. Finally, eight articles involving 1292 cases published from 2012 to 2020 meeting the inclusion criteria were enrolled [13, 16, 22–25] (Figure 1).

### 3.2. Study Characteristics

Five articles were carried out in Asia (including 1 in China, 1 in Israel, 2 in Korea, and 1 in Japan), 1 in Italy, and 2 in the USA. All articles were published from 2012 to 2020, with a sample size of 39–529. All the studies were retrospective. Six studies analyzed stage I NSCLC patients, and two studies analyzed stage I and II NSCLC patients. Three studies analyzed PFS, 1 analyzed DFS, 1 analyzed RFS, and 6 analyzed OS. The follow-up duration was 13.2–68 months. These eight articles involved at least one histological characteristic and treatment.  Table 1 presents details on all the enrolled articles, treatment, and histology. In addition, the FDG injection volume was 370–666 MBq.  Table 2 tabulates fasting duration, blood glucose test before injection, interval after injection, and threshold determination.

### 3.3. Literature Quality Evaluation

**Figure 2:**
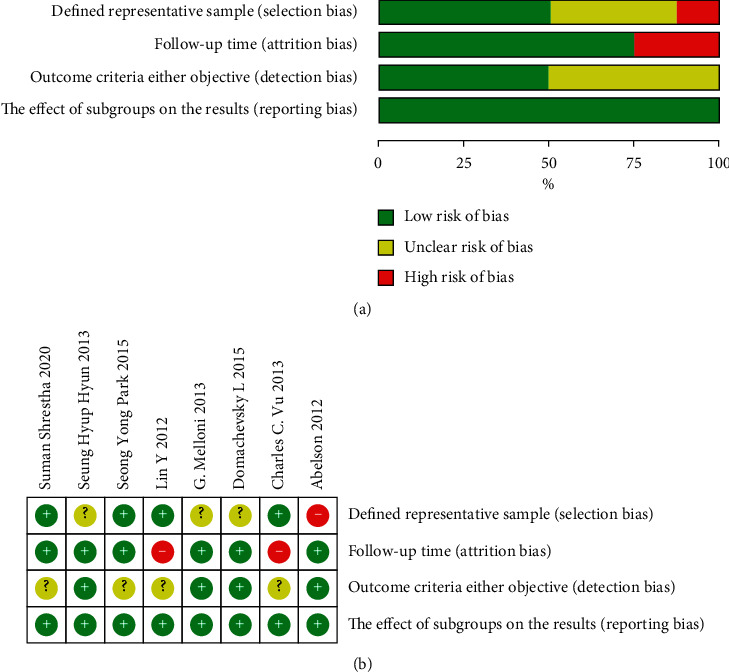
(a) Diagram showing the bias risk: judgment from researchers regarding the bias risk items shown in the form of percentages from the enrolled articles. (b) Summary of bias risk: judgment from researchers regarding the bias risk items from the enrolled articles.

This work evaluated all the enrolled study quality by CRITICAL APPRAISAL OF PROGNOSTIC STUDIES (https://www.cebm.net/wp-content/uploads/2018/11/Prognosis.pdf; Figure 2). The enrolled literature was carefully reviewed. Although the included studies were retrospective, most were high-quality. One of the enrolled articles was evaluated to be of high risk, while 3 of unknown bias risk in established typical sample measurement domain because of the nonrandomized or nonblinded study design. As for the prognostic factor domain, namely, the measurement of the follow-up period, two articles displayed a high bias risk, and 3 showed an unknown bias risk because median follow-up may not be long enough, and information on subsequent recurrences may be partially missing. Most enrolled articles were described well, and side reactions were observed objectively.

### 3.4. Primary Outcome: PFS

**Figure 3:**
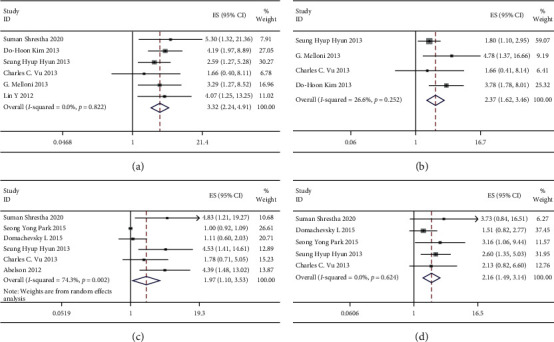
Forest plots of HR for PFS with MTV (a), TLG (b) and OS with MTV (c), TLG (d).

Five articles examined PFS and MTV. The HRs were combined, and the increased MTV value predicted poor PFS. No statistical significance was detected using the fixed-effects model (HR = 3.04; 95% CI = 1.92–4.81; *P* = 0.793; *I*^2^ = 0.0%) (Figure 3(a)), with no obvious heterogeneity across diverse articles. This study also carried out a sensitivity analysis to predict its influence on HRs. No obvious change was detected when a single study was eliminated in succession (Supplementary Figure 1(a)), which suggested result stability. Obvious publication bias was not detected from funnel plots (Supplementary Figure 2(a)). Egger's and Begg's tests were conducted to evaluate the possible publication bias. Neither Egger's (*P*=0.685) nor Begg's test (*P*=0.806) revealed obvious publication bias (Supplementary Figure 3(a)).

The chi-square test measures the heterogeneity. *P* < 0.05 is indicative of obvious heterogeneity. Squares = individual study point estimates. Horizontal lines = 95% CIs. Rhombus = summarized estimate and its 95%CI. Fixed: fixed-effects model. Random: random-effects model.

This study also conducted subgroup analyses stratified by the analysis, threshold, and region method (Table 3). In the region-stratified subgroup analysis, three articles from Asia showed HR of 3.22 (95% CI: 1.84–5.62; *P*=0.606), and one study from Europe showed significant correlations (HR = 3.29; 95% CI = 1.27–8.52). However, one study from America showed no significance (HR = 1.66; 95% CI = 0.44–8.11). One article adopting the ROC-based threshold method showed the HR value of 4.07 (95%CI: 1.25–13.25), while four adopting the threshold method based on additional methods showed HR of 2.89 (95%CI: 1.75–4.75; *P*=0.703). Concerning the analysis method, two articles according to multivariate analysis showed HR of 3.01 (95%CI = 1.59–5.67; *P*=0.370), while three based on univariate analysis showed HR of 3.08 (95%CI = 1.58–5.99; *P*=0.644).

Three articles examined PFS and TLG. The HRs were combined, which revealed that an increased MTV estimated a more dismal PFS. Statistical significance was detected from the fixed-effects model (HR = 2.02; 95% CI = 1.30–2.13; *P*=0.350; *I*^2^ = 4.7%) (Figure 3(b)), with no obvious heterogeneity among diverse articles. This study also conducted a sensitivity analysis to estimate the influence on pooled HRs. No obvious change was detected when a single study was eliminated in succession (Supplementary Figure 1(b)), indicating the stability of our results. Obvious publication bias was not detected from funnel plots (Supplementary Figure 2(b)). Due to only three studies being included, no potential publication bias and subgroup analyses were further assessed.

### 3.5. Secondary Outcome: OS

Six articles analyzed OS and MTV. The HRs were combined, and statistical significance was detected using the random-effects model (HR = 1.97; 95% CI = 1.10–3.53; *P*=0.002; *I*^2^ = 74.3%) (Figure 3(c)). However, sensitivity analysis for predicting the influence of pooled HRs was also conducted (Supplementary Figure 1(c)), which revealed no significance after the study of Suman Shrestha et al., Seung Hyup Hyun et al., or Abelson et al. was removed sequentially.

Five articles analyzed OS and TLG. The HRs were combined, and an increased TLG value was related to the dismal OS. Statistical significance was detected using the fixed-effects model (HR = 2.16; 95% CI = 1.49–3.14; *P*=0.624; *I*^2^ = 0.0%) (Figure 3(d)), with no obvious heterogeneity across diverse articles. A sensitivity analysis was also carried out for predicting the influence on pooled HRs, and no obvious change was detected when a single study was eliminated in succession (Supplementary Figure 1(d)), indicating the stability of our results. Obvious publication bias was not detected from funnel plots (Supplementary Figure 2(c)). Egger's and Begg's tests were conducted to assess the possible publication bias. Neither Egger's (*P*=0.216) nor Begg's test showed any obvious publication bias (Supplementary Figure 3(c)).

Further subgroup analysis was conducted by the analysis, threshold, and region method (Table 3). There were four articles in Asia, whose HR was 2.17 (95% CI: 1.46–3.23; *P*=0.455). One study in America did not reveal any significance (HR = 2.13; 95% CI = 0.75–6.04). There was 1 article adopting the ROC-based threshold method, which revealed no obvious significance (HR = 3.73; 95% CI = 0.84–16.51) and four studies adopting threshold method based on additional methods, which revealed significant correlation and HR of 2.09 (95%CI: 1.42–3.07; *P*=0.559). Three articles adopted multivariate analysis concerning the analysis method, whose HR was 2.85 (95%CI = 1.68–4.83; *P*=0.890). However, two studies using univariate analysis showed no significant correlations (HR = 1.65; 95% CI = 0.97–2.79).

## 4. Discussion

NSCLC cases are detected early. Therefore, it is crucial to estimate treatment outcomes or assess treatment response in the early stage. Our work focused on exploring the significance of 18F-FDG PET-derived MTV/TLG in predicting the prognosis of stage I/II NSCLC cases. TLG and MTV indicate the tumor biological features, thereby shedding light on tumor outcomes [26, 27]. Previous studies also provided prognostic information on PET for lung cancer. Im et al. [12] found that MTV and TLG on ^18^F-FDG PET were the typical factors to predict the prognosis of NSCLC cases. Jing et al. [11] discovered that the increased MTV and SUV max values from ^18^F-FDG PET/CT were related to a higher risk of relapse or mortality among the NSCLC cases receiving surgery. Eight studies included in total 1292 patients in this study, and different factors were found to affect TLG and MTV. As verified in this work, stage I/II NSCLC cases with increased TLG and MTV values were associated with a higher incidence of side reactions, whereas TLG was related to increased mortality risk. However, this work failed to illustrate the significance of MTV in predicting the mortality risk in stage I and II NSCLC patients.

Six articles analyzed MTV in OS. The HRs were combined, and the random-effects model (HR = 1.97; 95% CI = 1.10–3.53; *P*=0.002; *I*^2^ = 74.3%) (Figure 3(a)) showed statistically significant correlations. However, our results were not stable due to the small sample size revealed by sensitivity analysis, leading to poor statistical power. All six studies included can provide important prognostic information for stage I/II lung cancer. The study by Suman Shrestha et al., Seung Hyup Hyun et al., and Abelson et al. confirmed that high MTV was related to increased mortality risk. More large prospective articles could be conducted for validating MTV's significance in predicting the mortality risk for stage I/II NSCLC cases.

There was no evident heterogeneity detected for MTV in predicting PFS (*I*^2^ = 0.0%; *P*=0.793). Besides, Egger's and Begg's tests for MTV in PFS did not reveal any obvious bias of publication. However, some confounders might affect the relationship of MTV/TLG with survival. As a result, subgroup analysis was carried out by the analysis, threshold, and region method. In region-stratified subgroup analysis, Asian location, others group, and multivariate and univariate groups showed statistical significance and no heterogeneity. Only one American study analyzed PFS with MTV, which did not significantly correlate with each other (HR = 1.66; 95% CI = 0.44–8.1). Our result reliability was influenced by the not high enough statistical power. In this meta-analysis, PFS, EFS, and DFS were combined and redefined as PFS. Only Hyun et al. [15] analyzed DFS and Vu et al. [16] analyzed RFS. Thus, we did not perform additional subgroup analyses according to the endpoint. More articles are needed to validate MTV's prognosis prediction effect for stage I-II NSCLC patients.

Similarly, no evident heterogeneity was detected for OS in predicting TLG (*I*^2^ = 0.0%; *P*=0.624). Moreover, Egger's and Begg's tests for TLG in OS did not reveal any obvious bias of publication. However, some confounders might affect the relationship of MTV/TLG with survival. Subgroup analysis was carried out by the analysis, threshold, and region method. In region-stratified subgroup analysis, Asian location, others group, and multivariate group revealed no statistically significant heterogeneity. Only 1 study in the American subgroup (HR = 1.66; 95% CI = 0.44–8.1) and two studies of the univariate subgroup (HR = 1.65; 95% CI = 0.97–2.79) analyzed OS with TLG, which showed no significant correlations. The not high enough statistical power might affect our result reliability. Thus, more articles are needed to validate MTV's prognostic significance in stage I-II NSCLC patients.

No apparent heterogeneity was detected for PFS in predicting TLG (*I*^2^ = 43.2%; *P*=0.117) since only three studies analyzed PFS with TLG, which showed significant correlations. Sensitivity analysis supported that our results were stable. More studies are needed for validating PFS's prognostic value for TLG in stage I/II NSCLC patients.

MTV and TLG are both affected by SUV (standard uptake value) [18]. However, SUV is influenced by several patient-dependent and technical parameters, such as blood glucose levels, fasting duration, uptake duration, and attenuation correction, which must be strictly controlled [28]. Following the 18F-FDG PET imaging guidelines, the heterogeneity in PET/CT parameters was within normal limits (Table 2) [18, 29, 30]. SUV and other confounders possibly influence the relation of MTV/TLG with survival, and the increased TLG and MTV were related to patient survival. However, this study failed to establish the best threshold for MTV or TLG. Future high-quality study design and methods could find the best threshold for TLG and MTV.

However, our study had several limitations. First, all our enrolled articles were retrospective studies where results might not be robust enough, which may carry biases. Second, SUV or additional confounders may affect survival, MTV, and TLG. Besides, our study failed to determine the best threshold for MTV and TLG. Third, PFS, EFS, and DFS were not identical, which may lead to bias. Fourth, there may be language bias since it included only English-published studies. Additionally, follow-up time and selection of some works were high risks, leading to potential imprecisions. Nonetheless, evaluating publication bias supports our result reliability. Therefore, for further confirmation, more multicenter RCTs should be conducted.

## 5. Conclusion

Our work verified that stage I/II NSCLC cases with increased TLG and MTV have a higher risk of side reactions, and TLG is related to increased mortality risk. However, this work did not suggest that MTV significantly predicts the mortality risk in stage I and II NSCLC patients. More large prospective articles should be conducted to verify the significance of TLG and MTV in predicting the prognosis of stage I/II NSCLC cases.

## Figures and Tables

**Table 1:** Features of included studies.

Study	Year	Country	Study period	Follow-up duration (months)	Median age (range), years	No. of patients	TNM staging	End points	Study design	Histology	Treatment
Suman et al.	2020	Japan	2010.6–2016.10	44.8	73 (53–85)	39	I	OS PFS	Retro	Adenocarcinoma Squamous cell carcinoma	Radiotherapy
Domachevsky et al.	2015	Israel	2007–2012	60	68.7 ± 8.9	181	I-II	OS	Retro	Adenocarcinoma Squamous cell carcinoma Mixed/sarcomatoid Bronchioloalveolar Adenocarcinoma with bronchioloalveolar	Surgery
Seong et al.	2015	Korea	2006.2–2011.12	36.6	63.03 ± 10.01	248	I	OS	Retro	Adenocarcinoma Nonadenocarcinoma	Surgery
Seung et al.	2013	Korea	2003.7–2006.12	60	63 (23–83)	529	I-II	OS DFS	Retro	Adenocarcinoma Squamous cell carcinoma Large cell carcinoma Adenosquamous carcinoma Others	Surgery Chemotherapy Radiotherapy
Charles et al.	2013	USA	2007.5–2012.12	25.1	73.3	50	I	OS RFS	Retro	Adenocarcinoma Squamous cell carcinoma Others	Radiotherapy
Melloni et al.	2013	Italy	2005.1–2011.1	21 (3–68)	68(40–85)	99	I	PFS	Retro	Adenocarcinoma Others	Surgery
Abelson et al.	2012	USA	2005.9–2009.12	13.2	80.1(57.6–93.4)	84	I	OS	Retro	Squamous cell carcinoma Adenocarcinoma Non-small-cell Atypia	Radiotherapy
Lin Y et al.	2012	Taiwan	2009.1–2011.2	24(8–36)	63(38–85)	62	I	PFS	Retro	Squamous cell carcinoma Adenocarcinoma	Surgery

Abbreviations: Retro = retrospective; PFS = progression-free survival; DFS = disease-free survival; RFS = recurrence-free survival; OS = overall survival.

**Table 2:** 18F-FDG PET imaging methods for enrolled articles.

Study	Duration of fasting	Preinjection blood glucose test	Postinjection interval	Dose of 18F-FDG	Determination of cut-off values	Cut-off values	
						MTV(cm^3^)	TLG
Suman et al.	6 h	150 mg/dL	60 min	400 MBq	ROC	6.625	NA
Domachevsky et al.	NA	NA	NA	370–666 MBq	Others	7.1	NA
Seong et al.	6 h	140 mg/dL	60 min	5.5 MBq/kg	Others	7.3	8.8
Seung et al.	6 h	150 mg/dL	50 min	370 MBq	Others	16	70
Charles et al.	4–6 h	200 mg/dL	60 min	10–20 mCi	Others	NA	NA
Melloni et al.	6 h	180 mg/dl	60 min	370 MBq	Others	2.95	9.61
Abelson et al.	4–8 h	160 mg/dl	45–60 min	10–18 mCi	Others	NA	NA
Lin et al.	4 h	NA	45 min	370 MBq	ROC	9.8	NA

Abbreviations: ROC, receiver operating characteristic; MTV, metabolic tumor volume; TLG, total lesion glycolysis; NA, not available.

**Table 3:** Subgroup of PFS of MTV and OS of TLG.

Endpoint	Volumetric parameters	Factor	No. of studies	Heterogeneity test (*I*^2^, *P*)	Effect model	HR	95% CI of HR	Conclusion
PFS	MTV	Region						
		Asian	3	0.0, 0.606	Fixed	3.22	1.84, 5.62	Significant
		European	1	—	—	3.29	1.27, 8.52	Significant
		American	1			1.66	0.44, 8.1	Insignificant
		Cut-off method						
		ROC	1	—	—	4.07	1.25, 13.25	Significant
		Others	4	0.0, 0.703	Fixed	2.89	1.75, 4.75	Significant
		Analysis method						
		Multivariate analysis	2	0.0, 0.370	Fixed	3.01	1.59, 5.67	Significant
		Univariate analysis	3	0.0, 0.644	Fixed	3.08	1.58, 5.99	Significant

OS	TLG	Region						
		Asian	4	0.0, 0.455	Fixed	2.17	1.46, 3.23	Significant
		American	1	—	—	2.13	0.75, 6.04	Insignificant
		Cut-off method						
		ROC	1	—	—	3.73	0.84, 16.51	Insignificant
		Others	4	0.0, 0.559	Fixed	2.09	1.42, 3.07	Significant
		Analysis method						
		Multivariate analysis	3	0.0, 0.890	Fixed	2.85	1.68, 4.83	Significant
		Univariate analysis	2	0.0, 0.577	Fixed	1.65	0.97, 2.79	Insignificant

HR, hazard ratio; CI, confidence interval; PFS, progression-free survival; OS, overall survival; MTV, metabolic tumor volume; TLG, total lesion glycolysis; ROC, receiver operating characteristic.

## Data Availability

The data used to support the findings of this study are available from the corresponding author upon request.

## Supplementary Materials

Supplementary Materials Supplementary Figure 1. Sensitivity analysis for PFS with MTV (a), TLG (b) and OS with MTV (c), TLG (d). PFS = progression-free survival, OS = overall survival, MTV = metabolic tumor volume, TLG = total lesion glycolysis. Supplementary Figure 2. Funnel plots for PFS with MTV (a), TLG (b) and OS with TLG (c). The pseudo 95% confidence interval (CI) was computed as part of the analysis to produce the funnel plots and corresponded to the expected 95% CI for a given standard error (SE). HR indicates hazard ratio. PFS = progression-free survival, OS = overall survival, MTV = metabolic tumor volume, TLG = total lesion glycolysis. Supplementary Figure 3. Egger's test for PFS with MTV (a), TLG (b) and OS with TLG (c). The pseudo 95% confidence interval (CI) was computed as part of the analysis to produce the funnel plots and corresponded to the expected 95% CI for a given standard error (SE). HR indicates hazard ratio. PFS = progression-free survival, OS = overall survival, MTV = metabolic tumor volume, TLG = total lesion glycolysis. .
