# Supplementary figures and images for: Prognostic Value of MTV and TLG of 18F-FDG PET in Patients with Stage I and II Non-Small-Cell Lung Cancer: a Meta-Analysis
Source: Contrast Media Mol Imaging. 2021 Nov 22;2021:7528971. doi: 10.1155/2021/7528971 (PMC8629622; doi:10.1155/2021/7528971)

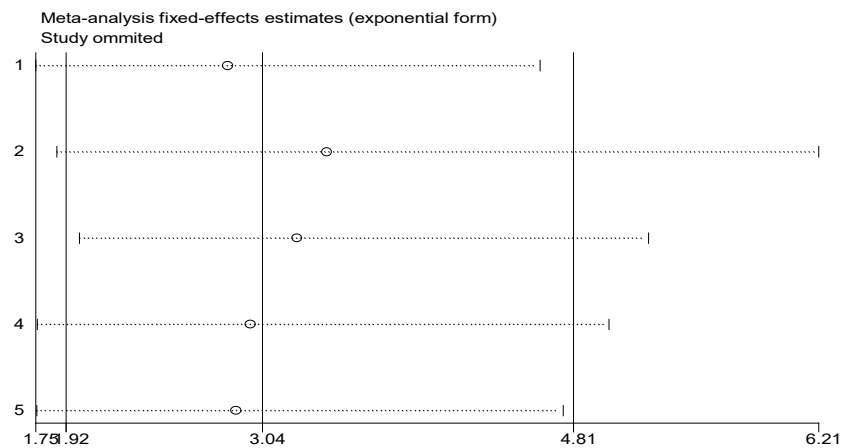

(a)

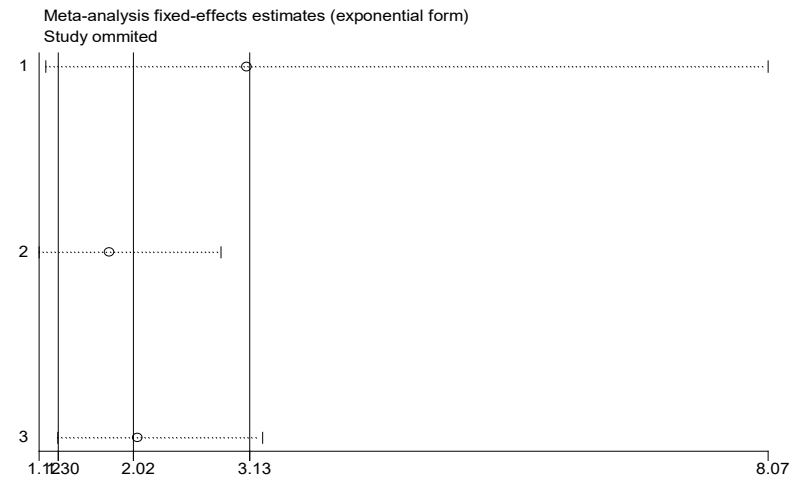

(b)

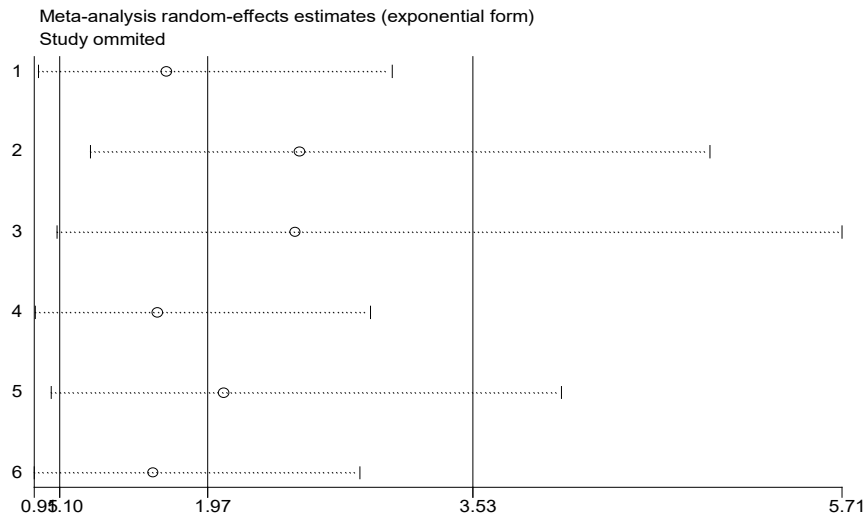

(c)

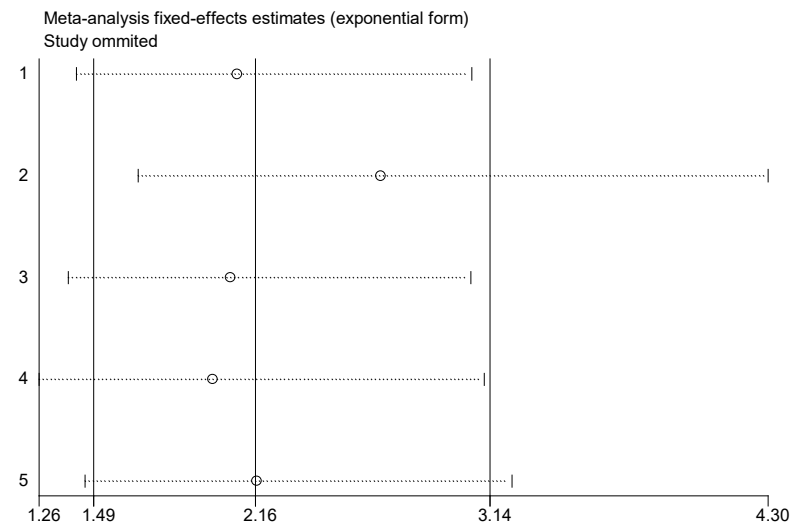

(d)

Supplement: Supplementary Materials — Supplementary Figure 1. Sensitivity analysis for PFS with MTV (a), TLG (b) and OS with MTV (c), TLG (d). PFS = progression-free survival, OS = overall survival, MTV = metabolic tumor volume, TLG = total lesion glycolysis. Supplementary Figure 2. Funnel plots for PFS with MTV (a), TLG (b) and OS with TLG (c). The pseudo 95% confidence interval (CI) was computed as part of the analysis to produce the funnel plots and corresponded to the expected 95% CI for a given standard error (SE). HR indicates hazard ratio. PFS = progression-free survival, OS = overall survival, MTV = metabolic tumor volume, TLG = total lesion glycolysis. Supplementary Figure 3. Egger's test for PFS with MTV (a), TLG (b) and OS with TLG (c). The pseudo 95% confidence interval (CI) was computed as part of the analysis to produce the funnel plots and corresponded to the expected 95% CI for a given standard error (SE). HR indicates hazard ratio. PFS = progression-free survival, OS = overall survival, MTV = metabolic tumor volume, TLG = total lesion glycolysis. . [file 7528971.f1.zip › 7528971.f1/Supplementary Figure 1.pdf]

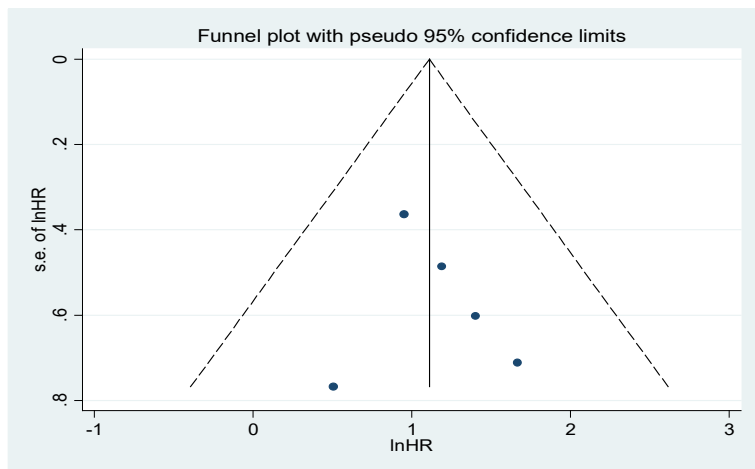

(a)

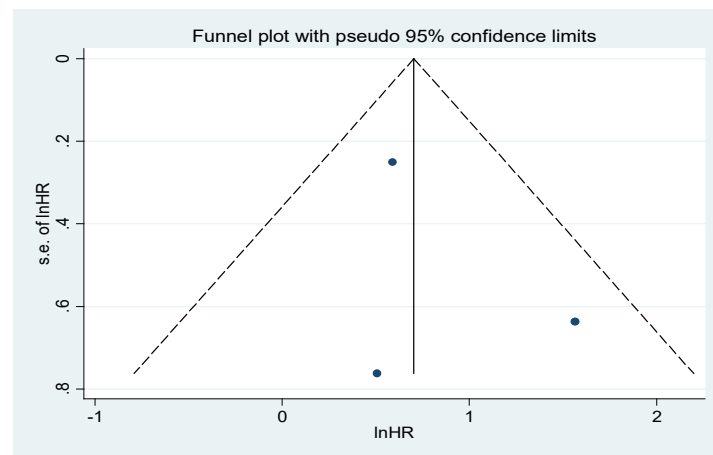

(b)

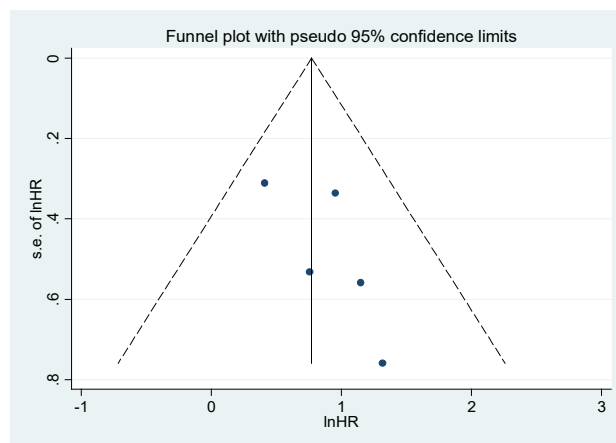

(c)

Supplement: Supplementary Materials — Supplementary Figure 1. Sensitivity analysis for PFS with MTV (a), TLG (b) and OS with MTV (c), TLG (d). PFS = progression-free survival, OS = overall survival, MTV = metabolic tumor volume, TLG = total lesion glycolysis. Supplementary Figure 2. Funnel plots for PFS with MTV (a), TLG (b) and OS with TLG (c). The pseudo 95% confidence interval (CI) was computed as part of the analysis to produce the funnel plots and corresponded to the expected 95% CI for a given standard error (SE). HR indicates hazard ratio. PFS = progression-free survival, OS = overall survival, MTV = metabolic tumor volume, TLG = total lesion glycolysis. Supplementary Figure 3. Egger's test for PFS with MTV (a), TLG (b) and OS with TLG (c). The pseudo 95% confidence interval (CI) was computed as part of the analysis to produce the funnel plots and corresponded to the expected 95% CI for a given standard error (SE). HR indicates hazard ratio. PFS = progression-free survival, OS = overall survival, MTV = metabolic tumor volume, TLG = total lesion glycolysis. . [file 7528971.f1.zip › 7528971.f1/Supplementary Figure 2.pdf]

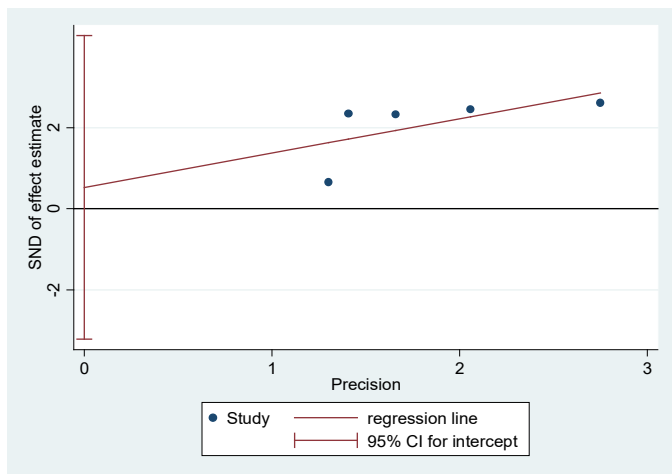

(a)

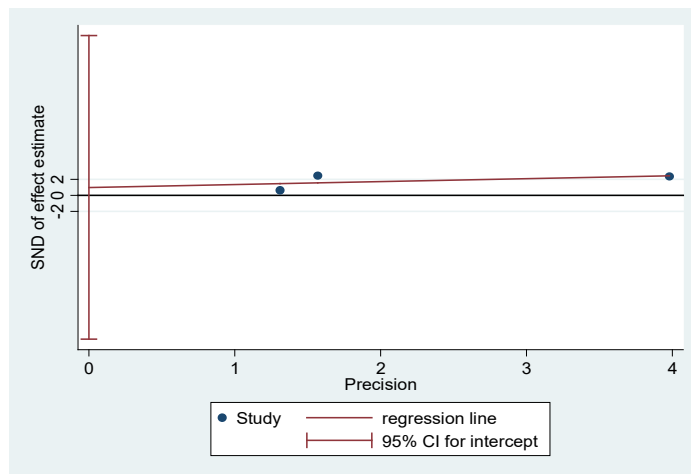

(b)

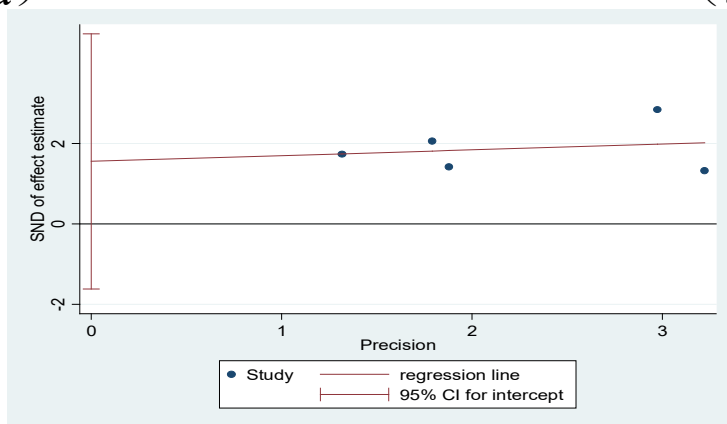

(c)

Supplement: Supplementary Materials — Supplementary Figure 1. Sensitivity analysis for PFS with MTV (a), TLG (b) and OS with MTV (c), TLG (d). PFS = progression-free survival, OS = overall survival, MTV = metabolic tumor volume, TLG = total lesion glycolysis. Supplementary Figure 2. Funnel plots for PFS with MTV (a), TLG (b) and OS with TLG (c). The pseudo 95% confidence interval (CI) was computed as part of the analysis to produce the funnel plots and corresponded to the expected 95% CI for a given standard error (SE). HR indicates hazard ratio. PFS = progression-free survival, OS = overall survival, MTV = metabolic tumor volume, TLG = total lesion glycolysis. Supplementary Figure 3. Egger's test for PFS with MTV (a), TLG (b) and OS with TLG (c). The pseudo 95% confidence interval (CI) was computed as part of the analysis to produce the funnel plots and corresponded to the expected 95% CI for a given standard error (SE). HR indicates hazard ratio. PFS = progression-free survival, OS = overall survival, MTV = metabolic tumor volume, TLG = total lesion glycolysis. . [file 7528971.f1.zip › 7528971.f1/Supplementary Figure 3.pdf]
